# Predictors of long-acting contraceptive utilization hot spots in Ethiopia: using geographical weighted regression analysis

**DOI:** 10.3389/fgwh.2024.1420476

**Published:** 2024-08-12

**Authors:** Hailemichael Kindie Abate, Abere Woretaw Azagew, Chanyalew Worku Kassahun, Mulugeta Wassie, Chilot Kassa Mekonnen, Yilkal Abebaw Wassie, Alebachew Ferede Zegeye

**Affiliations:** Department of Medical Nursing, School of Nursing, College of Medicine and Health Sciences, University of Gondar, Gondar, Ethiopia

**Keywords:** long-acting contraceptive, geographically weighted regression, predictors, contraceptive, Ethiopia

## Abstract

**Background:**

The use of long-acting contraceptives is a common health challenge in Ethiopia. Therefore, the current study aimed to assess the determinants of using long-acting contraceptive hot spots in Ethiopia using data from the Ethiopian Mini Demographic and Health Survey for 2019.

**Methods:**

This study used data from the Ethiopia Mini Demographic and Health Survey 2019 and included a total weighted sample of 8,885 women in the analysis. The geographical variation of long-acting contraceptive usage was initially observed using hot spot analysis. Arc GIS version 10.7 was used for geographically weighted regression. Ordinarily, least squares regression was performed to identify predictors that explain the geographical variation in the use of long-acting contraceptives. Geographic weighted regression was used to predict the hot spot area of long-acting contraceptive methods.

**Results:**

The overall prevalence of long-acting contraceptive utilization use was 6.9% (95% confidence interval: 6.4–7.45). Most of the statistically significant hot spots for long-acting contraceptives were found in lactated areas of the Oromia part of Amhara and Dire Dawa. Primary education, followers of the Muslim religion, marital status, and women with >4 children were the determinants of spatial variation use of hot spot areas for long-acting contraceptive methods.

**Conclusions:**

A detailed map of long-acting contraceptive use hot spots and their determinants will enable decisions to target their sociodemographic-related predictors of women.

## Introduction

The long-acting contraceptive (LAC) method is among the most suitable and effective modern contraceptive methods that can prevent pregnancy and save money for families and the country as a whole (1). Long-acting contraceptives can be reversible or non-reversible. The reversible type of contraceptive can be hormonal (implants that prevent pregnancy for 3–5 years) and non-hormonal [an intrauterine device (IUD) that prevents pregnancy for 12 years]. The permanent type of contraceptive (vasectomy/sterilization of the man and tubal ligation of the woman) can prevent pregnancy for life (1).

In addition to the prevention of pregnancy, contraceptives have other uses, such as for adjusting the menstrual cycle, controlling premenstrual mood disturbances, and preventing endometrial cancer (1–3).

In developing countries, approximately 214 million women of reproductive age lack access to modern contraception techniques, including LAC methods. As a result, nearly half of pregnancies are unintended (4). In addition, one in three unwanted pregnancies is the consequence of contraceptive failure, which is more common with traditional and short-acting techniques than with long-acting ones (5).

Ethiopia is one of the countries with the fastest population growth, next to Nigeria (6). According to evidence from the Ethiopia Demographic Health Survey (EDHS) of 2016, 22% of women still lack access to contraceptive methods (7). Providing appropriate, accessible, and inexpensive contraceptive options is crucial to prevent unwanted pregnancies and help families achieve their desired health goals (5). Globally, 64% of women of reproductive age used different methods of contraception, of which approximately 45.2% of women in developed countries used long-acting contraceptive methods. Of them, 5.1% were in sub-Saharan Africa (8, 9). In Ethiopia, most women of reproductive age used short-acting contraceptive methods and only 10% of the women used LACs (10, 11).

To expand the lack of access to contraceptive methods, the Ethiopian Ministry of Health must plan to distribute LAC to prevent unintended pregnancy at all healthcare levels (12). Despite these attempts to increase contraceptive utilization, there was still a low level of contraceptive utilization (11).

Studies conducted on long-acting contraceptives in Ethiopia showed the simple prevalence of the utilization of long-acting contraceptives and its associated factors. As research evidence, the age of the women (13, 14), marital status, level of education (15, 16), wealth index (16, 17), number of children (18–20), and residency were factors in the use of long-acting contraceptives (17, 21). Studies were conducted on the geographical variation of modern contraceptives among reproductive age groups using data from the 2019 EDHS (22, 23). However, no studies were conducted on the determinants of long-acting contraceptive utilization hot spot analysis using 2019 mini-EDHS data applying geographic weight regression. Therefore, the current study aimed to assess the determinants of the utilization of long-acting contraceptive hot spot analysis in Ethiopia using 2019 mini-EDHS data.

## Methods

### Study setting, data source, and study period

**Figure 1:**
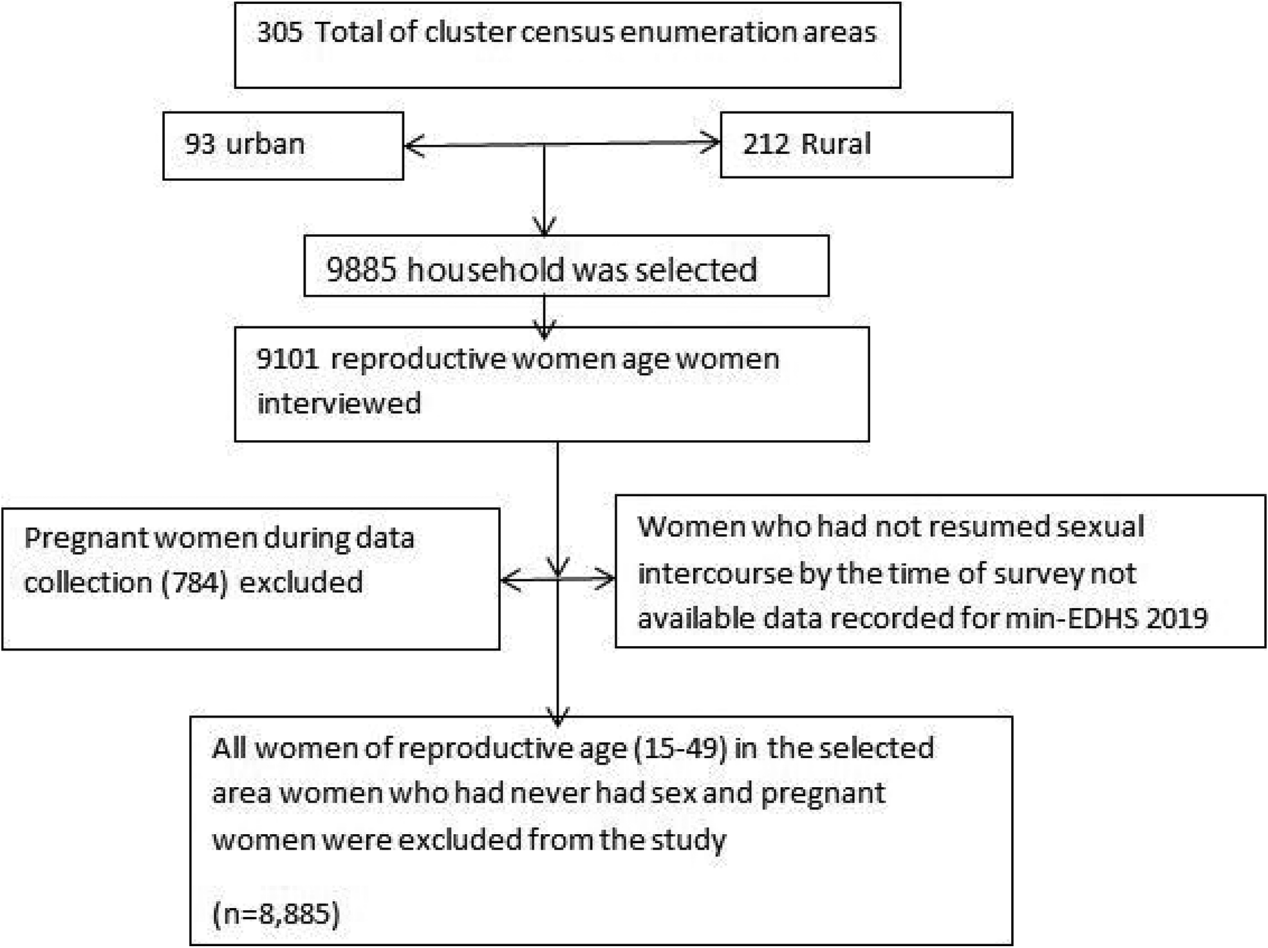
The flow chart of women using LAC.

The study was carried out in Ethiopia, which is located in the Horn of Africa, 3° to 14° N and 33° to 48° E. The Ethiopia Mini Demographic Health Survey (EMDHS) 2019 data were used as a data source for this study (24). According to EMDHS 2019, there are nine administrative regions [Tigray, Afar, Amhara, Oromia, Benishangul-Gumuz, Gambela, Southern Nations, Nationalities and People's Region (SNNPR), Harari, and Somoli] and two city administrations (Addis Ababa and Dire Dawa). EMDHS 2019 was the second mini-demographic survey conducted in Ethiopia. The sample was stratified into two stages and selected. A total of 305 cluster census enumeration areas (EA) stratified into urban and rural (93 urban and 212 rural) areas were selected (Figure 1).

### Study population, eligibility criteria, and study period

The study was carried out between 21 March 2019 and 28 June 2019. All women of reproductive age in their late 20s were the source population, while those selected reproductive-age women who were in their late 20s were the study population. All women of reproductive age in the selected area were included in the study, while women who had never had sex and pregnant women were excluded.

### Sampling procedures

In total, 8,885 women were included in the study. The dataset used to analyze this manuscript was an individual record (IR). The detailed sampling procedure is illustrated in the 2019 EMDHS report.

### Study variables

#### Dependent variable

The dependent variable of this study was the use of one of the long-acting contraceptives (used/not used) (25).

#### Independent variables

The individual-level factors included were age, marital status, religion, educational status, wealth index, number of living children, and age at first birth, while community-level factors included community wealth index, residency, region, and community education (26, 27).

### Operational definition

#### Long-acting contraceptive method

Women who are considered to be using long-acting contraceptives use one of the following long-acting contraceptive methods: IUD, implants, and male/female sterilization (25).

#### Community wealth index

The community wealth index is calculated by generating the combined wealth index with its cluster number. Then, it was leveled as high community poverty and low community poverty by computing the median of the proportion (0.2777778) of the wealth index of women.

#### Community education level

The educational level of the participants that is considered a high-level educational status of the community is generated using the media proportion of the highest educational status (0.9220659) of the participants.

### Data management and analysis

Microsoft Excel was used to clean up the data. The data were further processed, cleaned, recoded, and analyzed using STATA version 14/MP. Before spatial analysis, the sample waiting for the outcome variable and the explanatory variable was performed to ensure the representativeness of the DHS data sample and to obtain reliable estimates and standard errors (28). Descriptive data were analyzed using STATA and presented in tables to describe the use of long-acting contraceptives by sociodemographic factors and maternal characteristics. STATA version 14/MP and Arc GIS v.10.8 were used for the data analysis.

### Spatial analysis

The spatial autocorrelation of Global Moran's I measure was computed to assess the spatial heterogeneity of the use of long-acting contraceptives. Moran's I values close to negative 1 indicate dispersed, close to positive clustered, and 0 values indicate random use of long-acting contraceptives (29). A significant statistical Moran I value (*p* < 0.05) led to the rejection of the null hypothesis, which indicated the presence of spatial autocorrelation. The hot spot analysis (Getis–Ord statistics) of the z scores with significant *p*-values gave the characteristics with hot spot or cold spot values for the clusters. The Getis–Ord statistic z score near zero indicates that there is no clustering, the positive z score indicates a cluster of high value (hot spot), and the negative z score indicates a cluster of low value (cold spot) (30).

### Spatial regression

Ordinary least squares (OLS) regression is a global statistical model that uses a single equation to estimate the relation between the outcome and explanatory variables. The equation is given as $\gamma_{i}=\beta_{i\;}+\Sigma_{k=1\;}^{p}\beta_{k}x_{ik}+\varepsilon_{i}$ where *i* = 1, 2, … *n*, *β*_0_, *β*_1_, *β*_3,_ …, *β_P_* are the model parameters, *Y_i_* is the outcome variable for the observation *i*, *x_ik_* is the explanatory variable, and ℰ_1_, ℰ_2_, ℰ_3_, …, ℰ*_n_* are the error term/residuals with zero mean and with homogenous variance $\sigma^{2}$.

After identifying long-acting contraceptive hot spots, spatial regression modeling was performed to identify predictors of spatial patterns of long-acting contraceptives. The ordinary least squares finding is only reliable if the regression model satisfies all the assumptions that are required by this method (31). All OLS assumptions, such as multicollinearity using the variance inflation factor (VIF), the Koenker test for non-stationarity, Moran's I statistics for residual spatial autocorrelation, and statistically non-significant Jarque–Bera statistics for model bias, were checked before preceding the geographically weighted regression (GWR).

GWR is a local statistical regression model that assumes cluster variation (non-stationarity) in the relationship or heterogeneity in the relationship between the dependent and independent variables in different cluster areas. In contrast, OLS fits a single linear static equation to all data in the study area, while GWR enables an equation for each enumeration clustered area (32, 33). Therefore, the GWR coefficient assumes various values for each cluster. The coefficients were associated with each explanatory variable in the maps that were generated using the GWR. The equation of GWR was given as $\gamma_{i}=\beta_{0}(u_{i}\;v_{i})+\sum_{k=1}^{p}\beta_{k\;}(u_{i},v_{i})x_{ik\;}+E$, where *γ*_i_ is the observation’s response *γ*, (*u_i_v_i_*) are the geographical points of latitude and altitude, *β*_k_(*u_i_v_i_*) (*k* = 0, 1, 2, …, *p*)*p* is the unknown function of geographic location (*u_i_v_i_*), *X_ik_* is the explanatory variable at location *u_i_v_i_*, *i* = 1. 2, 3, …, *n*, and $E_{i}$ is an error terms with zero mean and homogenous variance. To minimize potential confounders’ variable with *p*-value > 0.2 in OLS had to be excluded for GWR analysis.

### Ethical approval

Permission to access the data was obtained from a Demographic Health Survey (DHS) through an online request at https://dhsprogram.com/data/available-datasets.cfm. The data used for this study were freely available on request online. This study was based on secondary data from the EDHS, and we obtained a permission letter from the main Demographic Health Survey.

## Results

Among the total study participants, the majority (95.05%) of Muslim women did not use long-acting contraceptives. Among women who had not used long-acting contraceptives, the majority (98.59% and 94.18%) were not married and had no educational level, respectively. The age of respondents at first delivery (98.2%) and participants who have no living children (90.31%) did not use log-acting contraceptive methods (Table 1).

**Table 1:** The weighted proportion of sociodemographic factors in the use of long-acting contraceptive methods (*n* = 8,885).

Variables	Categories	Utilization of long-acting contraceptive		Total
		Used, *N* (%)	Not used, *N* (%)	
Religion	Orthodox	459 (13.60)	3,074 (86.40)	3,374
	Catholic	4 (6.41)	74 (93.59)	78
	Protestant	126 (7.36)	1,585 (92.63)	1,711
	Muslim	180 (4.95)	3,455 (95.05)	3,635
	Other	4 (4.60)	83 (95.4)	87
Marital status	Married	568 (10.12)	5,045 (89.88)	5,613
	Not married	46 (1.41)	3,226 (98.59)	3,272
Educational level	No education	212 (5.82)	3,428 (94.18)	3,640
	Primary	255 (7.62)	3,090 (92.38)	3,345
	Secondary	73 (6.35)	1,076 (93.65)	1,149
	Higher	74 (9.85)	677 (90.15)	751
Age of respondent at first birth (years)	<20	416 (9.69)	3,878 (90.31)	4,294
	≥20	157 (10.12)	1,395 (89.88)	1,552
Wealth status	Poor	149 (4.42)	3,223 (95.58)	3,372
	Middle	107 (8.44)	1,161 (91.56)	1,268
	Rich	358 (8.43)	3,887 (91.57)	4,245
Number of living children	None	43 (1.38)	3,070 (98.2)	3,113
	1–2	274 (11.82)	2,045 (88.18)	2,319
	3–4	160 (9.73)	1,485 (90.27)	1,645
	>4	137(7.58)	1,671(92.42)	1,808

Table 2 illustrates the weighted proportion of long-acting contraceptive use according to region and place of residence. Among women who had not used long-acting contraceptives, 93.4% were rural residents of the country. Similarly, most of the participants (99.52%) in the Somali region and 96.7% in the far region do not use the long-acting contraceptive method (Table 2). The overall prevalence of long-acting contraceptive use was 6.9% (95% confidence interval: 6.4–7.45).

**Table 2:** The weighted proportion of long-acting contraceptive use by place of residence and region, EMDHS 2019.

Variables	Utilization of long-acting contraceptive			*P*-value
	Used, *N* (%)	Not used, *N* (%)	Total	
Residency				
Urban	459 (7.60)	5,565 (92.40)	6,024	<0.01
Rural	190 (6.64)	2,671 (93.36)	2,861	
Region				
Tigray	69 (10.97)	560 (89.03)	629	<0.01
Afar	2 (2.35)	83 (97.65)	85	
Amhara	171 (8.40)	1,856 (91.60)	2,026	
Oromia	232 (6.93)	3,115 (93.07)	3,347	
Somali	2 (0.48)	419 (99.52)	421	
Benishangul-Gumuz	13 (13.13)	86 (86.87)	99	
SNNPR	112 (6.57)	1,593 (93.43)	1,705	
Gambela	1 (2.49)	40 (97.51)	41	
Harari	2 (7.40)	25 (92.60)	27	
Addis Ababa	41 (9.28)	401 (90.72)	442	
Dire Dawa	5 (7.70)	60 (92.30)	65	

### Spatial autocorrelation and significant hot spots of long-acting contraceptive utilization

**Figure 2:**
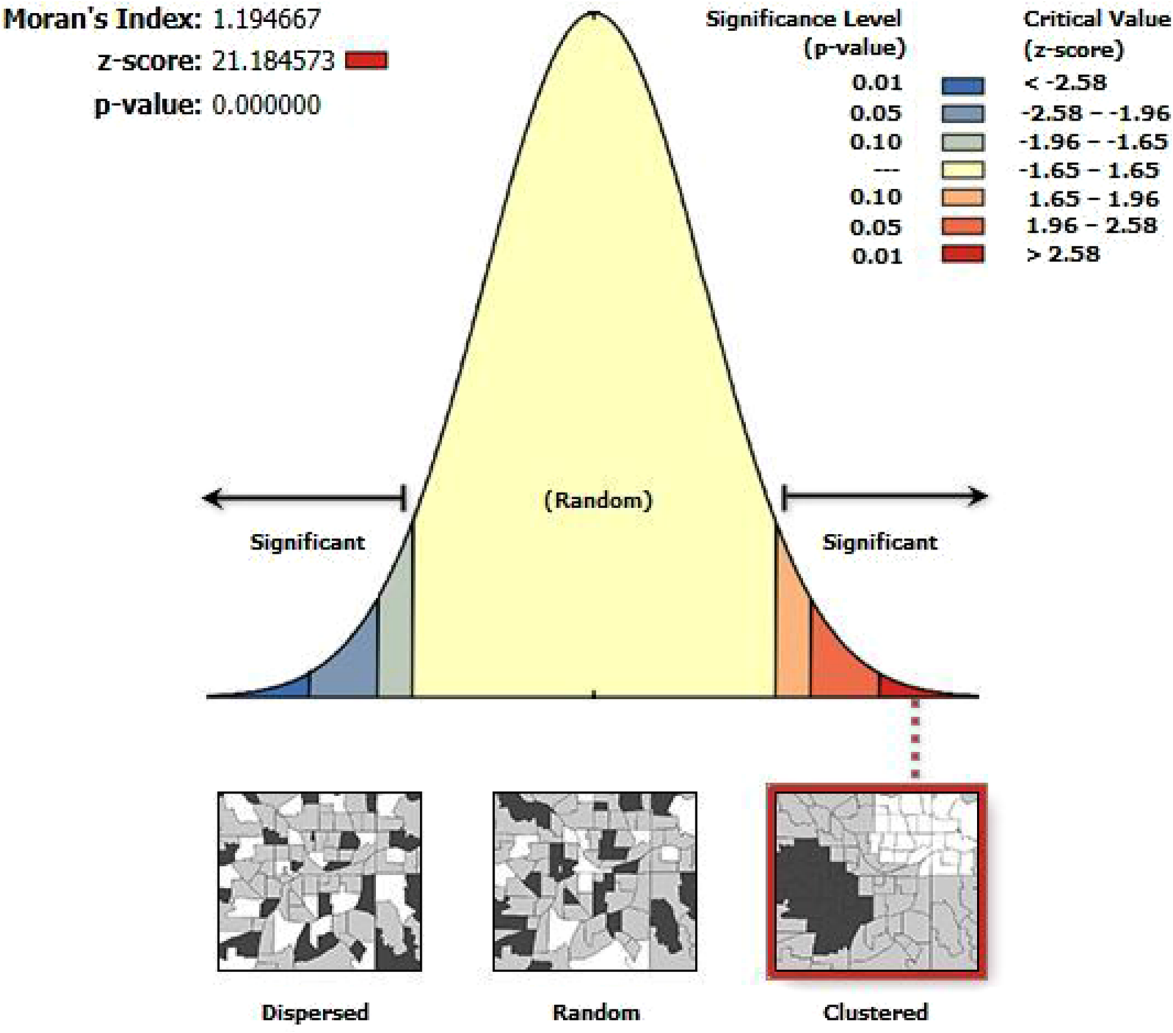
The global spatial autocorrelation analysis of LAC usage among women aged 15–49 years in Ethiopia, EMDHS 2019.

The spatial autocorrelation analysis showed a significant spatial autocorrelation of the use of long-acting contraceptives throughout the country with the Global Moran I value of 1.19 (*p*-value < 0.01) (Figure 2).

**Figure 3:**
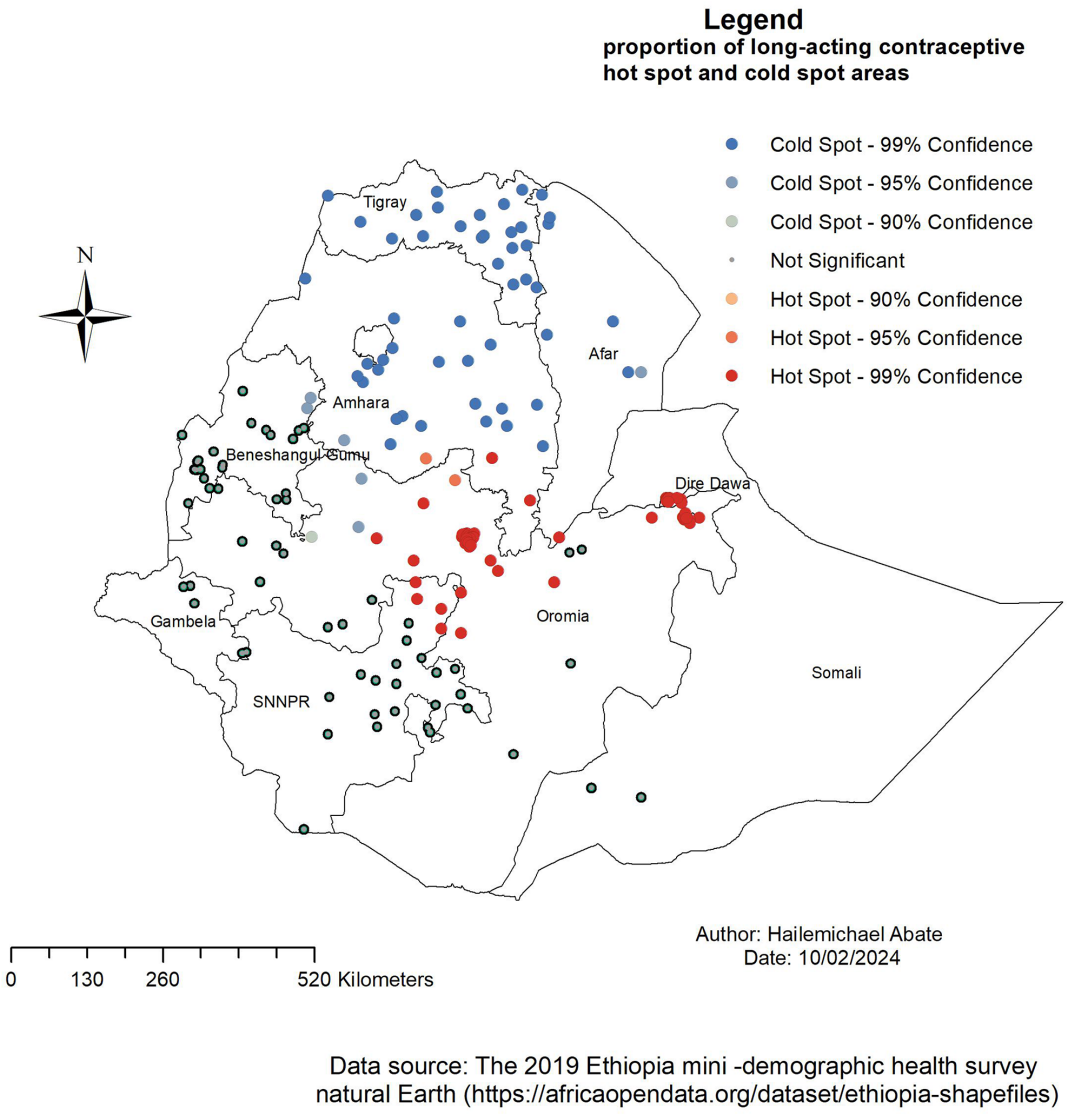
The Getis–Ord Gi statistical analysis of LAC usage hot spots among women aged 15–49 years in Ethiopia, EMDHS 2019.

The significant hot spots for long-acting contraceptive use were lactated in the Oromia part of Amhara, Dire Dawa, and SNNPR (Figure 3).

### Factors affecting the spatial variation of LAC utilization

The result of the OLS model showed that 16% of the variation in LAC utilization (adjusted R^2^ = 0.16) was explained by the model and met all assumptions of the OLS statistical model (34). There was no multicollinearity between the independent variables, that is, the VIF was less than 10. The Koenker test for non-stationarity was statistically significant (*p*-value < 0.01); therefore, it is recommended to perform a geographical weight regression. Since the Jarque–Bera statistic was non-significant (*p*-value > 0.01), the residual was assumed to be normally distributed. Furthermore, the combined Wald statistic was statistically significant (*p*-value < 0.01), which indicates that the overall model was significant. As a result of the meeting of all the OLS assumptions, GWR had to be run to obtain the local coefficient of explanatory variables of the hot spot areas (Table 3).

**Table 3:** Model summary of OLS for significant variables of LAC utilization in Ethiopia, EMDHS 2019.

OLS diagnostics			
Number of observations	202	AIC	1,395.28
Multiple R^2^ (d)	0.17	Adjusted R^2^	0.16
Joint F-statistic	4.74	Prob(>F), (8,193) degrees of freedom	0.000025*
Joint Wald statistic	41.01	Prob(>chi-squared), (8) degrees of freedom	0.000002*
Koenker (BP) statistic	22.13	Prob(>chi-squared), (8) degrees of freedom	0.004670*
Jarque–Bera statistic	84.52	Prob(>chi-squared), (2) degrees of freedom	0.08

* *p* < 0.05, statistically significant.

The proportion of followers of the Muslim religion, mothers with a primary education, married women, and women with >4 children had a significant influence on the distribution of the use of LAC. A unit increase for respondents with a primary education and marital status increases the use of long-acting contraceptives by 0.122 and 0.16, respectively. However, there was a unit decrease in followers of the Muslim religion and women with >4 children by −0.047 and −0.09 in the use of LACs, respectively (Table 4).

**Table 4:** Analysis results of OLS for LAC utilization in Ethiopia, EMDHS 2019.

Variable	Coefficient	Standard error	t-statistics	Probability	Robust standard error	Robust t-statistics	Robust probability	VIF
Intercept	0.35	0.52	0.068	0.049	0. 48	0.073	0.046	—
Muslim religion	−0.047	0.013	−3.46	<0.01	0.012	−3.69	<0.01	1.08
Mother with primary education	0.122	0.038	3.21	<0.01	0.043	2.80	<0.01	1.12
Married status	0.16	0.044	3.646	<0.01	0.043	3.70	<0.01	1.81
Women having >4 children	−0.09	0.042	−2.140	0.033	0.036	−2.51	0.012	1.31

### Geographical weighted regression analysis

The OLS statistical regression model showed the LAC factors in the geographical hot spot area. However, it was a global model that showed that the relationship between each explanatory variable and LAC is stationary in the study area. The results of the GWR indicated that there was a significant improvement compared to OLS. In Table 4, the adjusted R^2^ (0.16) and Akaike's Information Criterion (AIC) (1,395.28) obtained in OLS were increased in GWR to 0.17 and 1,394.93, respectively. Based on this analysis, the GWR result is better than the result generated using the OLS model (Table 5).

**Table 5:** Analysis result of significant explanatory variables using the GWR model for LAC in Ethiopia, EMDHS 2019.

Explanatory variables	Women with primary education, Muslim religion, richest wealth quartiles, women having >4 children
Residual squares	10,428.71
Effective number	12.20
Sigma	7.41
AIC	1,394.93
Multiple R^2^	0.18
Adjusted R^2^	0.17

The GWR analysis results showed that the explanatory variables were weak and had a strong relationship with the use of LAC.

**Figure 4:**
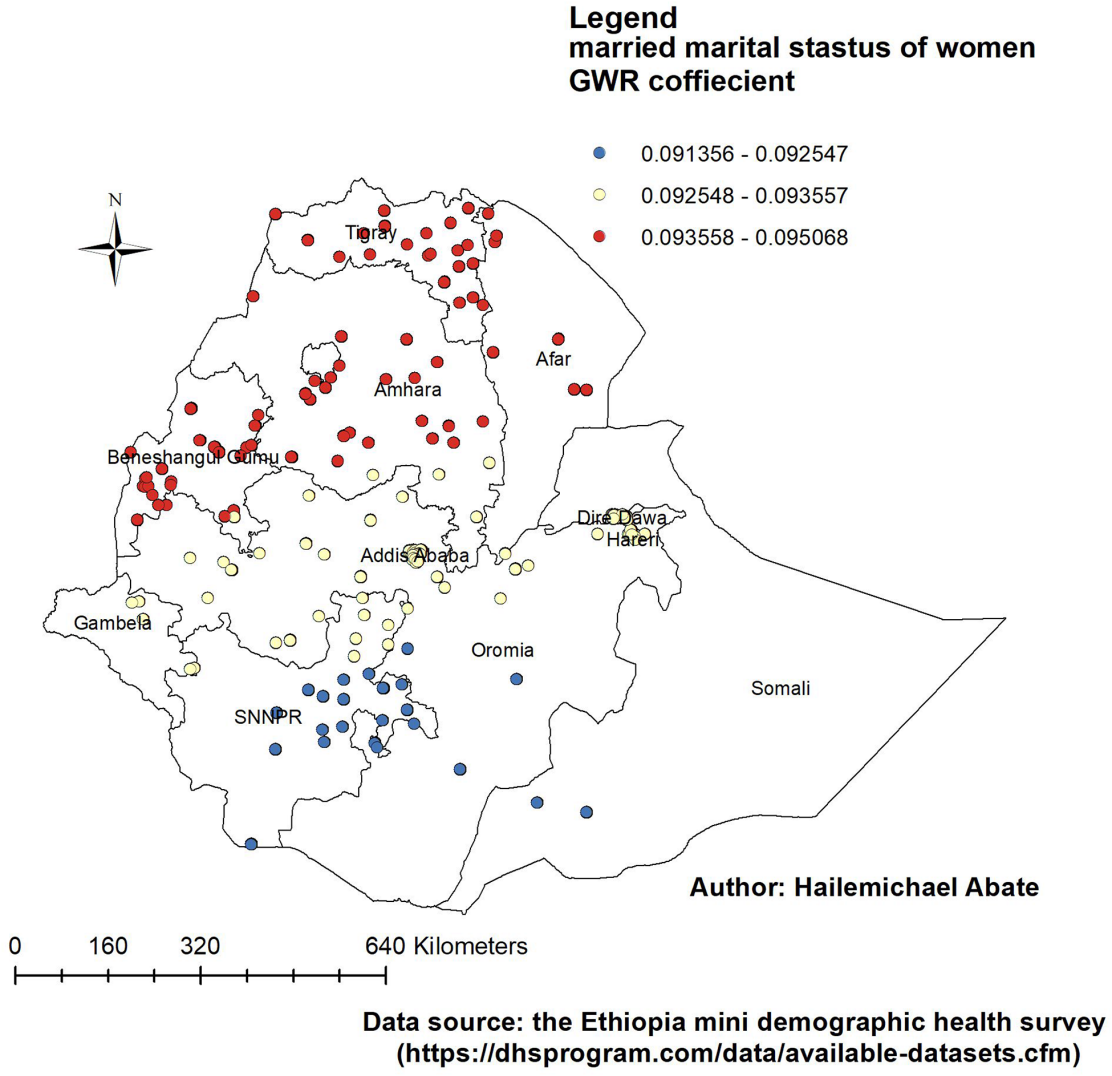
Women with GWR coefficients of married marital status to predict LAC usage in Ethiopia, EMDHS 2019.

**Figure 5:**
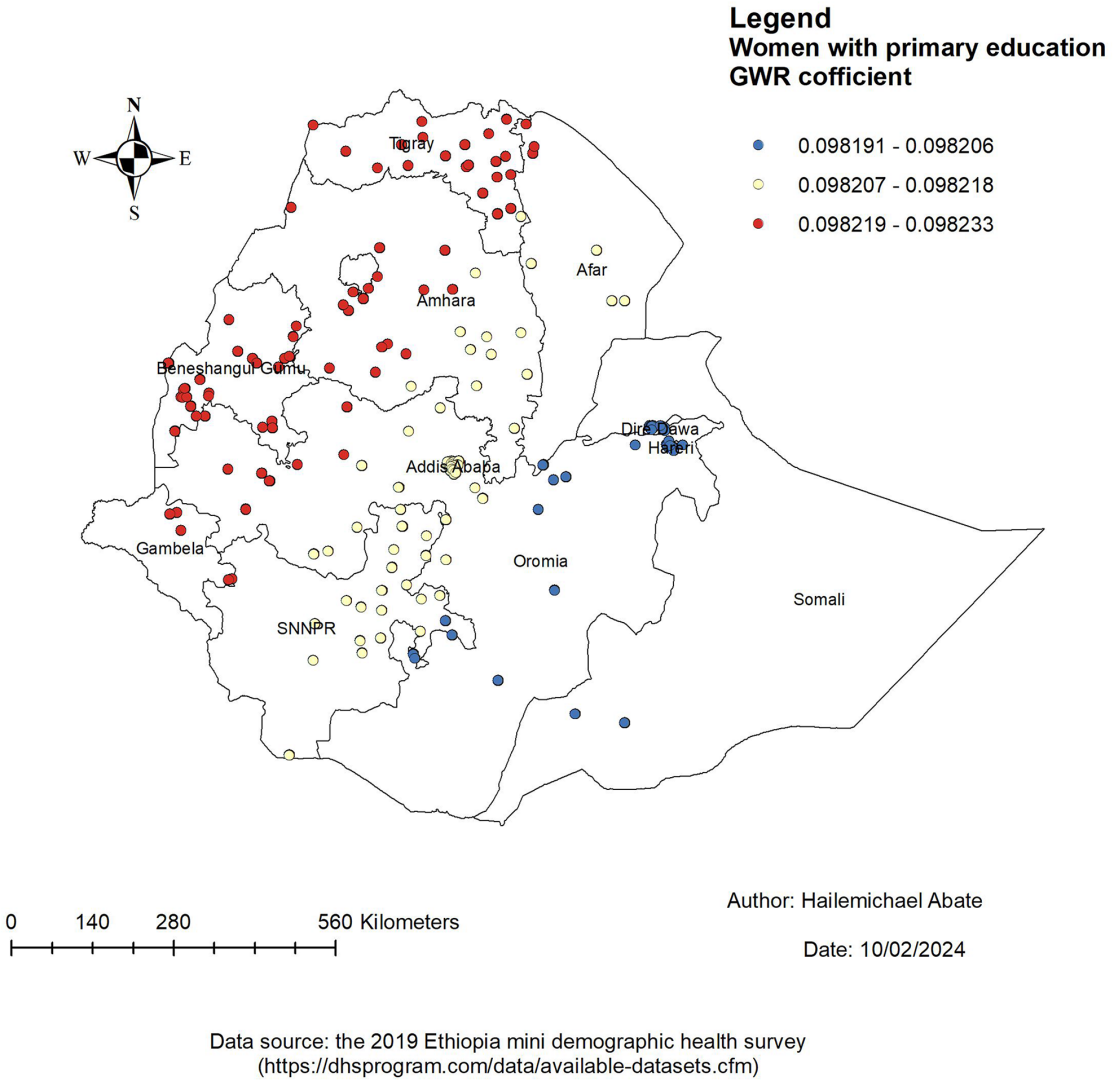
Women with GWR coefficients of primary education to predict LAC use in Ethiopia, EMDHS 2019.

**Figure 6:**
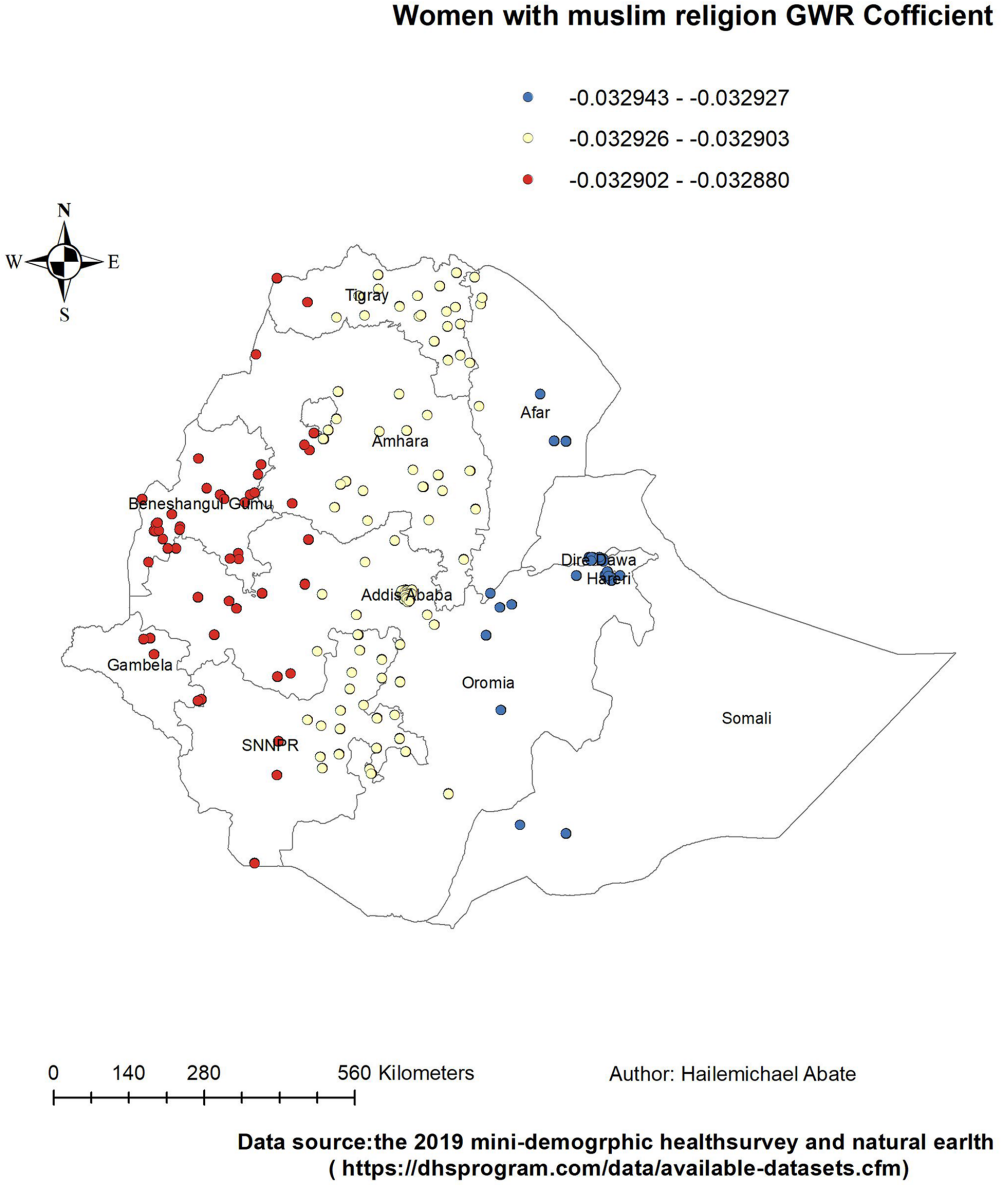
Women with GWR coefficients of Muslim religion to predict LAC usage in Ethiopia, EMDHS 2019.

**Figure 7:**
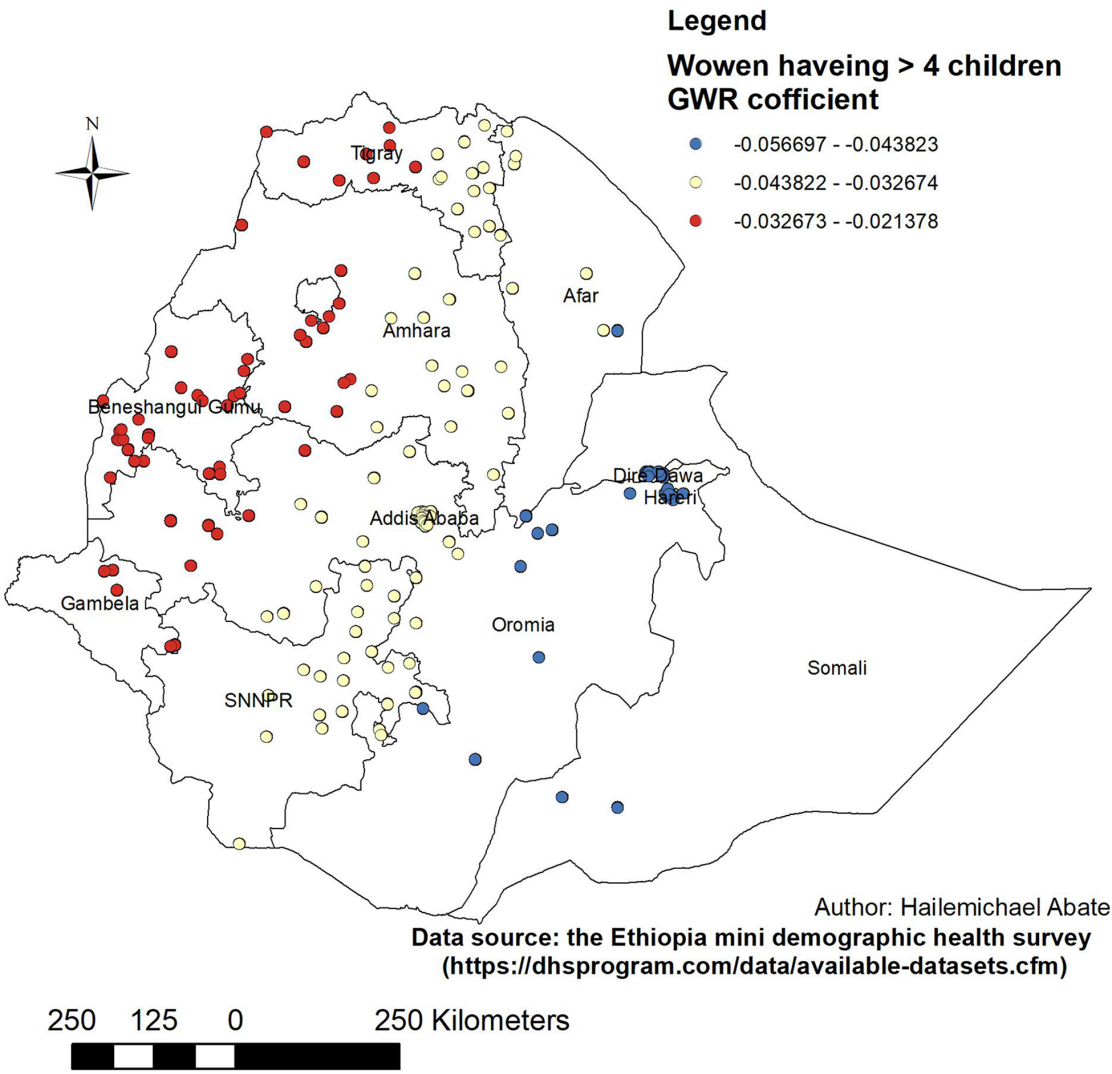
Women with GWR coefficients of >4 children to predict LAC usage in Ethiopia, EMDHS 2019.

Figures 4–7 show that the proportion of explanatory variables of LAC in the geographical areas was strong and weak predictors. The geographical area with clustered red points indicates the largest coefficient of the predictors and strong predictors in the hot spot areas in the use of long-acting contraceptives. Therefore, the largest coefficient of the predictor in the hot spot areas indicates a strong predictor in the use of LAC. Women who were married had strong and positive predictors of LAC. As the proportion of women who were married increased, the incidence of LAC usage in Tigray, Amhara, Benishangul-Gumuz, and parts of Afar increased (Figure 4).

Women with a primary education had a positive and weak predictor of LAC. As the proportion of women with a primary education increased, the existence of LAC in Tigray, Amhara, Benishangul-Gumuz, Gambela, west Oromia, and the northern part of Afar increased (Figure 5).

Women who were followers of the Muslim religion had a negative and weak relationship with the use of LAC. Therefore, the Muslim religion was a protective factor in the use of LAC hot spot areas. The proportion of Muslim women decreased in the LAC hot spot areas of West Amhara and Oromia; in the entire Benishangul-Gumuz; and in parts of Tigray, Gambela, and SNNPR (Figure 6).

In the same way, women with >4 children had a negative and weak relationship with the use of LAC. Therefore, the factor of women with >4 children was protective in the use of LAC hot spot areas. The proportion of women with >4 children decreased in the LAC hot spot areas of Tigray, Amhara, Gambela, Benishangul-Gumuz, northwest Tigray, southwest Amhara, and parts of Gambela (Figure 7).

## Discussion

This baseline study shows the geographical variation of LACs and their associated factors in Ethiopia. LAC hot spots were located in Tigray, Amhara, Benishangul-Gumuz, Afar, Tigray, Oromia, SNNPR, and Gambela. Based on the findings of this study, the potential disparity in the use of LACs across the region could be due to different sociodemographic factors. In the GWR analysis, statistically significant determinant factors are identified that had strong and weak relationships with the use of LACs.

The proportion of women who were married, who had a primary education, who were Muslim, and who had >4 children were the predictors of the geographic areas of the LAC hot spots. From these predictors, the GWR coefficient of primary education relatively indicated that strong predictors of LAC were in the range of 0.098219–0.098233. The findings of this study can be used to emphasize the distribution use of LAC in the least used area of Ethiopia by addressing the predictors in the use of LAC.

The GWR coefficient of those women who were married varied from region to region in the range of 0.0938558–0.095068. The proportion of women who were married in the state increased the incidence of LAC use in Tigray, Amhara, Benishangul-Gumuz, and parts of Afar. This study's findings differed from the studies conducted in the 2016 EDHS (27, 35) in Ghana (36). A possible justification might be because of the difference in use of the dataset and method of analysis. Furthermore, participants in the current study might have knowledge in the use of LAC during pregnancy follow-up visits, the exchange of information with their couples, and the participation of husbands in reproductive services (37, 38).

The finding of this study also indicated that the proportion of women who had a primary education had a positive relationship and increased the prevalence of LAC use in Tigray, Amhara, Benishangul-Gumuz, Gambela West, and the northern part of Afar. This finding was supported by studies conducted in Ethiopia (39–41), Nigeria (42), and sub-Saharan Africa (43). A possible explanation might be that educated women would have information about the importance of the use of LAC through different social media and reading different articles (44, 45).

However, religious Muslim women had a negative and weak relationship with the use of LAC. Therefore, the Muslim religion was a protective factor in the use of LAC hot spot areas. The proportion of religious Muslim women decreased in the use of LAC hot spots area of West Amhara and Oromia, in the entire Benishangul-Gumuz, and in some parts of Tigray, Gambela, and SNNPR. The finding in this study is consistent with studies conducted in Ethiopia (46), Nigeria (47), and Nepal (48). A possible justification could be that religious opposition to contraceptive use may be more problematic for Muslim women (49).

Similarly, women who had >4 children had a negative and weak relationship with the use of LAC. Therefore, the factor of women who had >4 children was protective in the use of LAC hot spot areas. The proportion of women who had >4 children decreased in the use of LAC hot spot areas of Tigray, Amhara, Gambela, Benishangul-Gumuz, northwest Tigray, southwest Amhara, and parts of Gambela. This finding is supported by the study conducted in sub-Saharan Africa (50). This could be because women who had more children could be approaching physiological menopause and thus experience decreased sexual desire so there is no need for the use of long-acting contraceptives (51).

The present study has some limitations. The geographical coordinate cluster area was displaced by 2 km in urban areas, 10 km for 1% of the clusters in rural areas, and 5 km for most of the clusters in rural areas to avoid anonymized respondents of the community. This may influence the estimated cluster effects in the spatial analysis. Furthermore, in the two EMDHS 2019, some maternal factors were not available; this could be a potential determinant of LAC. However, despite these limitations, the study also had its strengths. The study used a nationally representative dataset that covers all regions of the country. In addition, the spatial regression modeling analysis using GWR was the strength of this study.

## Conclusions

The study showed that there was spatial variation in the use of long-acting contraceptives among women in Ethiopia. Significant hot spot areas of long-acting contraceptive use were lactated in Oromia, part of Amhara, Dire Dawa, and SNNPR.

Women who were married and had a primary education had a positive predictor of LAC. On the other hand, religious Muslim women and women with >4 children had a negative relationship with the use of LAC.

Decision makers and the government should emphasize in women’s health information about the use of contraceptives and cooperate with religious and community leaders as strategies to improve the use of long-acting contraceptives. Organizing campaigns with religious leaders on the use of contraceptives is a good strategy to improve the use of contraceptives by women. In addition, education about the factors that influence the negative attitude toward the use of contraceptives is another effective means to increase the use of contraceptives. Furthermore, researchers should investigate other cultural and social factors that hinder the geographical variation of the use of long-acting contraceptives.

## Data Availability

The original contributions presented in the study are included in the article/Supplementary Material, further inquiries can be directed to the corresponding author.
